# Editors-in-chief in social sciences: Mapping the institutional, geographical, and gender representation between academic fields

**DOI:** 10.1371/journal.pone.0317931

**Published:** 2025-02-20

**Authors:** Manuel Goyanes, Luis de-Marcos, Timilehin Durotoye, Triwik Kurniasari, Homero Gil de Zúñiga

**Affiliations:** 1 Department of Communication, Carlos III University of Madrid, Getafe, Spain; 2 Universidad de Alcalá, Alcalá de Henares, Madrid, Spain; 3 The Pennsylvania State University, University Park, Pennsylvania, United States of America; 4 University of Salamanca, Salamanca, Spain; 5 Universidad Diego Portales, Santiago, Chile; University of Siena, Italy, ITALY

## Abstract

This study systematically maps the network structure of the editors-in-chief in social sciences journals, focusing on their gender representation, geographical distribution, and institutional composition. Drawing upon large-scale data from 3,320 JCR-ranked journals of 57 different fields in the social sciences (4,868 editors-in-chief from 1,485 affiliations of 71 countries), the study aims to illustrate the current connections of editorial leadership in social sciences. Findings reveal that two countries—the U.S. and the U.K.—and their institutions shape almost all fields of the social sciences, with institutions from other geographies, particularly non-English-speaking countries, being substantially underrepresented. However, there is no central institution that dominates across all fields, but within dominant geographies, a reduced number of different affiliations prevail in the most important intellectual terrains. In terms of gender representation, there is a significant imbalance across all dimensions under study. Male editors-in-chief outnumber females across most fields (66.67%), countries (76.60%), and affiliations (63.16%). All in all, by critically mapping the connections of editors-in-chief in social sciences journals, this study seeks to advance our understanding of the current structure of editorial governance and, in turn, stimulate initiatives aimed at fostering a more representative leadership in social science, keeping levels of scientific excellence constant.

Within the marketplace of scholarly publishing in social sciences, the composition/representation [1,2], background [3,4], and effects [5,6] of editorial boards in research output stand as a crucial [7], yet relatively uncovered area of academic inquiry. Within this specific strand of literature, the examination of the connection between the leadership agents (i.e., editors-in-chief or EiCs) is even more surprising, considering their paramount role in the decision-making process of peer review and in setting the research agendas, thematic patterns, and methodological approaches of academic journals [8,9]. In this context, while the examination of editorial boards in general and EiCs emerges as an established area of research to understand power dynamics in science [10], a notable gap persists in our understanding regarding the gender, institutional, and geographical connections surrounding the editorial leaders of academic journals in social sciences. In this study, we seek to address this gap in the literature by undertaking a large-scale analysis of the connections, gender dynamics, institutional, and geographical representation of EiCs within all disciplines of social sciences covered by the Journal Citation Reports (JCR) ranking.

Generally, the scientometric literature on meta-research has largely focused on understanding the content, methodological [11,12], and authorship [13,14] patterns of scholarly publications across social science fields, rather than the individual agents guiding the editorial governance. Studies on editorial boards, however, have been prolific but rather specific, as the vast majority of previous studies have mainly focused on the educational background and training of EiCs [15], the efforts and challenges in promoting diversity and inclusion of editorial boards (including EiCs) [16,17], ethical concerns [18] and, more generally, the editorial decision-making processes namely, how EiCs and editorial board members make decisions on manuscript acceptance or rejection and what factors influence such decisions [19–21].

Our research aims to transcend the mere identification of the EiCs of journals across all social science subjects. Instead, it will focus on the network of connections that establish the power and governance dynamics in the leadership of social sciences. In this regard, existing studies often overlook the gender, institutional, and geographical footprint of editorial boards. By addressing this phenomenon, our investigation seeks to descriptively portray the subtle yet influential ties that weave the network of editorial leadership in social sciences journals.

As prior research has systematically observed, gender imbalances persist as a prevalent concern within academia, with a growing recognition of the need for equitable representation in leadership roles [22,23]. While strides have been made in acknowledging and addressing gender disparities in the different levels of sciences (rank, authorship, editorial board membership, and editorial leadership, among others) [24,25], it remains unknown the gender distribution of EiCs in most social science disciplines, beyond rather specific fields [26–29]. Our research endeavors to bridge this gap by investigating gender representation and theoretically exploring the possible implications. Besides gender dynamics, the institutional and geographical compositions of EiCs represent additional underexplored territories [10].

This paper, therefore, seeks not only to provide insights into the current state of editorial governance in social sciences journals but also to articulate a call for a more general (rather than field-specific) approach to studying the composition of EiCs. In doing so, we aim to contribute valuable insights to ongoing discussions on diversity and research pluralism within academia. Specifically, the study provides a descriptive account of the connections between all fields of social sciences by examining the roles and relationships of their editors-in-chief. We also offer a representation of the network structure encompassing the countries, institutions, and gender of these editors. Accordingly, our study is primarily descriptive, aiming to present a broad overview of the individuals who control the vast majority of journals within the social sciences and to propose some theoretical, albeit speculative, implications based on the findings.

Although academic disciplines differ in their focus, the potential affiliations shared by editors-in-chief may hold significance in understanding academic power dynamics and the influence these affiliations exert on global editorial decisions. Such decisions include maintaining the quality of a journal’s research output, which informs theory building, scientific inquiries, and scholarly practices [30,31]. While we do not claim that country- or affiliation-based intellectual commonalities directly shape the norms and values underlying the scientific cosmovision of all editors from the same country or institution, these factors may nonetheless leave an imprint on their decisions and expectations. In this regard, although the nature of our data cannot capture the extent of such influence, if any, it does allow us to identify key patterns of geographical and affiliation-based dominance, providing insights into the current status quo. That said, the aim of this network-based approach to studying editors-in-chief is not to provide empirical findings regarding their potential influence on what and how is published (see [6], for insights in the field of Communication). Rather, it seeks to offer a descriptive analysis of the editors’ network structure, paving the way for future studies to build upon this work and employ content analysis or other methodological approach to more accurately examine their potential influence on the expectations, methods, theoretical approaches, or statistical techniques used. Consequently, based on the above-mentioned objectives, the study is guided by the following research questions:

RQ1) What are the connections between all fields of social sciences characterized by the affiliation and country of the journals’ EiCs? At a more descriptive level, the study also seeks to address the country, affiliation, and gender level contribution to scientific fields, thus posing the following two research questions:
RQ2) What is the representation and network structure of countries/institutions of affiliation among EiCs in each social science field?
RQ3) What is the gender representation of EiCs within a) countries, b) affiliations, and c) scientific fields?

## Method

### Data collection

This study considered all 3,548 journals listed in the Social Science Citation Index (SSCI) of the JCR in the year 2021. For each journal, we gathered the name and category from the JRC. The JCR classifies SSCI journals under 57 categories, which represent the main research field for each journal. For journals listed in more than one category, we considered the first category listed by the JCR. Data of EiCs was manually collected from the web page of each journal. For this study, the EiCs were the ones clearly listed by each journal using a variety of names like EiC, chief editor, lead editor, general editor, editor, etc. In light of the selection methodology employed by [32], which also sets a threshold of 5 EiCs for journal analysis, this study considers only journals with 5 or fewer EiCs, ensuring a clear delineation of roles and responsibilities within the editorial leadership. Fifty-three journals did not report any EiC, and 175 reported more than 5 sEiCs. The final dataset included 4,868 EiCs reported by 3,320 journals. For each EiC, we collected the name, institutional affiliation, and country of affiliation as reported on the web page of the journal. For EiCs reporting more than one affiliation, we coded the first affiliation. In 53 cases, the affiliation of the EiCs was not reported, so their affiliation and country were coded as missing. For another 7 EiCs, the affiliation was reported as either a world organization (e.g., the World Health Organization) or a general role (e.g., *the Economist*), but it was not possible to determine the country because it was not reported. In such situations, the country was coded as missing. The final dataset included EiCs from 1,485 different affiliations in 71 countries.

Finally, the gender of each EiC was manually determined by the coders either using the name and picture when available on the web page of the journal or by searching in the reported institution. 2,959 EiCs were coded as male (60.78%), 1,843 as female (37.86%), and 66 were coded as missing (1.36%) because it was not possible to find them or determine their gender.

### Analytical tools

In addressing the concern regarding the relationship between EiCs, it is important to note that EiCs typically do not hold simultaneous roles across multiple journals. Furthermore, the cross-presence of editors across multiple boards, a phenomenon known as interlocking, has been substantially explored in existing literature [10,33–35]. This study contributes to the discourse by examining the projected relationships between EiCs, specifically focusing on how these relationships are shaped by the shared affiliations and countries of EiCs. The role of the EiCs is crucial in shaping the academic norms and habitus within the field of knowledge production, which can be influenced by both formal and informal societal structures [36]. The geographical representation of editorial boards also plays a significant role, as it can affect the diversity and number of publications within a journal, with a noted correlation between the regions of chief editors and the assignments of editorial boards [37]. Therefore, this study is focus on the relations between chief editors, underpinned by shared national and institutional backgrounds. This approach provides a nuanced understanding of the dynamics at play in academic journal governance and the broader landscape of scholarly communication.

We used the dataset collected in the previous step to build two different bipartite graphs that represented the connections between EiCs, affiliations, and countries. A bipartite network is a graph whose nodes are divided into two sets, and every edge connects a node of the first set to a node of the second set. Bipartite graphs are also called two-mode networks. The first network built for this study was the bipartite graph of fields-affiliations, weighted by the number of scholars that act as EiCs for each given affiliation and field. We constructed the bipartite network using a computer program. Initially, all fields and affiliations were added to the graph. Then, for each editor, a new edge was added to the graph connecting the field of the journal and the affiliation of the editor. If the edge was already in the graph because a previous EiC shared the same field and institution, then the weight of the edge was increased by one. The second network was an equivalent bipartite graph of fields-countries that connects fields and countries through their EiCs. The network was built in an analogous way, initially adding all fields and countries, and creating or updating the edges for each EiC. We used slices to determine the main subgroup of activity for both bipartite graphs. An *n*-slice is a subgraph that contains only the edges with weight equal to or larger than *n*.

To analyze the relations between entities of the same type, we used projections. A projection is a one-mode network including nodes of only one of the original sets, which are connected if they share a common neighbor in the original bipartite graph. Projections can be weighted by the number of shared neighbors. For this study, we constructed four weighted projections: one of countries, one of affiliations, and two of fields. The field projections represent connections based on shared affiliations and shared countries, respectively. As projections include only the connections between nodes of the same set, they facilitate visualizing direct relations between countries, affiliations, and fields. All projections were weighted by the number of EiCs that characterize the relation. For instance, in the projection of countries, two countries are connected if they share at least one EiC in any field, and the total number of EiCs shared in all fields is stored in the edge that connects the two countries. We also utilized slices to determine the main groups of activity at the different levels that the projections represented: fields, country, and institution.

In this study, we computed the following metrics for each node of the two main bipartite networks as well as for all nodes of the four projections: degree, closeness centrality, betweenness centrality, and clustering coefficient. Degree is the number of connections of each node. Closeness centrality is a measure of the position of a node in terms of distance to all other nodes. Higher centrality means that the node is closer to the center of the network. Betweenness centrality is a measure of the number of shortest paths that go through a given node. A high value means that the actor is in a brokerage position, having faster access to more information and connecting parts of the network that may otherwise be distant or unconnected. The clustering coefficient is a measure of the level of embeddedness of a node in its local neighborhood. A high value means that close nodes tend to cluster together, forming tighter local areas of activity. All metrics were normalized to the range 0–1.

Since the interpretation of metrics for the bipartite networks may not be straightforward, here, we provide it for the metrics of the fields in the field-country network. The interpretation for countries and for the other projection (field-affiliation) is analogous. Specific interpretations are given in the Results and Discussion sections when pertinent. A field’s degree in this network represents the number of countries that have at least one EiC associated with a journal in that field. A higher degree for a field suggests that EiCs from a wider range of countries are represented in that field’s journals. This could indicate a more globalized or internationally collaborative research environment. Conversely, a lower degree could imply a more geographically concentrated network of scholars within that field. Closeness centrality measures the average distance from a field to all other countries in the network, considering paths through shared EiC connections. A high closeness centrality score for a field indicates it is, on average, closer to other countries in the network. This could suggest the field is more integrated within the broader network of international scholarly communication, potentially leading to greater visibility and influence. A lower closeness centrality score might indicate a field is more isolated or has stronger ties to a smaller subset of countries. Betweenness centrality quantifies the number of shortest paths between any two countries that pass through a particular field node. In this context, a field with high betweenness centrality likely attracts EiCs from a diverse set of countries, some of whom might not otherwise be connected within the network. High values of betweenness centrality in this field-network indicate that the field serves as a point of connection among various countries through shared EiCs. This suggests that the field has the potential to facilitate scholarly communication across different national contexts. However, it is important to note that this metric does not imply that the EiCs actively engage in dialogue or collaboration with one another. Instead, it reflects the structural position of the field within the network, highlighting its role in linking diverse scholarly communities. The clustering coefficient measures the extent to which countries that share EiCs with a specific field also share EiCs among themselves. A high clustering coefficient for a field indicates that the countries represented by its EiCs tend to form a tightly-knit community. This could suggest shared research interests, regional collaborations, or a tendency for scholars from certain countries to gravitate towards particular fields. Conversely, a low clustering coefficient might suggest a more geographically dispersed network of EiCs within that field, possibly indicating a broader range of research influences.

Similarly, as the interpretation of the metrics for the four projections is not straightforward, so we provide here a more detailed one for the projection of affiliations. The interpretation for the other three projections (countries, fields through affiliations, and fields through countries) follows the same logic. In the projection of affiliations, each node represents an academic institution. An edge connects two institutions if they share at least one EiC across any social science field within the dataset. The weight of an edge signifies the total number of EiCs shared by those two institutions across all fields. An institution’s degree in this projected network represents the number of other institutions with which it shares at least one EiC. A higher degree suggests that the institution’s EiCs are connected to a broader array of other institutions through their editorial roles across various social science fields. This could indicate a wider network of potential collaborations and influence within the global research community. Conversely, a lower degree may imply a more limited network, potentially reflecting a narrower research focus or less international collaboration. Closeness centrality measures the average distance from one institution to all others in the network, considering the shortest paths through shared EiC connections. An institution with high closeness centrality is, on average, closer to other institutions in the network. This central positioning could facilitate faster dissemination of research, greater access to diverse perspectives, and potentially a higher degree of influence within the broader social science community. Betweenness centrality quantifies how often an institution lies on the shortest path between any two other institutions in the network. High betweenness centrality suggests a strategic position as a bridge or connector between institutions that might not be directly linked. This highlights a potential role in fostering interdisciplinary research, facilitating international collaborations, and influencing the flow of knowledge across different academic communities. The clustering coefficient of an institution measures the extent to which other institutions connected to it through shared EiCs are also connected to one another. A high clustering coefficient suggests that the institutions connected through shared EiCs tend to form a closely-knit community. This could indicate shared research interests, regional collaborations, or a tendency for these institutions to attract scholars with similar research profiles. Conversely, a lower clustering coefficient may suggest a more diverse and less interconnected network of affiliations.

We applied the Mann-Whitney U test with an adjustment for the number of editors as a covariate to compare gender differences in network metrics for fields, affiliations, and countries in which one gender was predominant. This non-parametric test is appropriate for comparing two groups (male vs female) while controlling for the effect of the number of editors when dependent variables are non-normally distributed, as is the case of our data. To do so, we computed a balance ratio for each field, affiliation, and country, labeling it as ‘male’ when the ratio of male to female was larger than 1.2 or ‘female’ when the ratio of female to male was larger than 1.2. The 1.2 threshold indicates that the majority group has 20% more members than the other. It was not possible to compute the ratio for countries and institutions with only one EiC. The balance ratio of the complete dataset is 1.61, and it was also used to show graphically the intensity of the prevalent group for each field, country, and affiliation in figures. We used the Python NetworkX package to create the networks and projections, and to compute the metrics because it provides implementations for bipartite networks. We used the Gephi software to produce the visualizations and figures with the Force-Atlas 2 layout.

## Results

### Geographical and institutional representation (RQ1 and RQ2)

**Fig 1:**
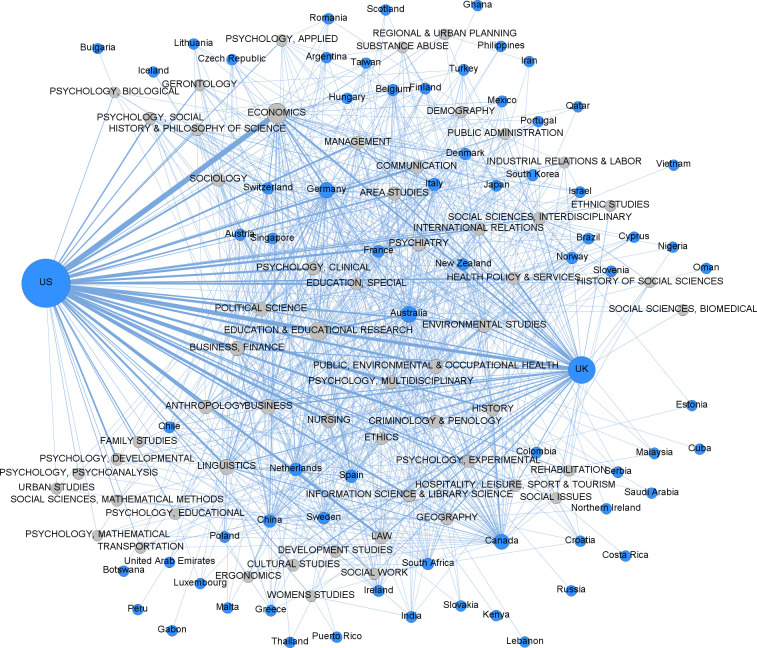
Bipartite graph of fields and countries, illustrating the connections between fields and the countries of affiliation of their Editors-in-Chief. Node size is proportional to the number of editors. Line thickness is proportional to the number of Editors-in-Chief in that field affiliated with institutions in the connecting country.

**Fig 2:**
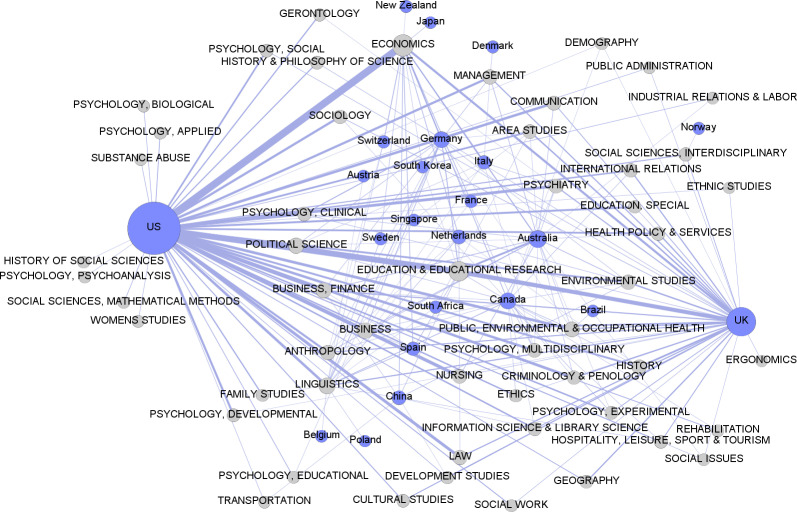
5-slice of the bipartite graph of fields and countries, showing only the connections with five or more Editors-in-Chief. Size of nodes is proportional to the number of editors. Line thickness is proportional to the number of Editors-in-Chief in that field affiliated with institutions in the connecting country.

In the bipartite network of countries and fields (Fig 1), fields are grouped into two main areas. One area includes fields related to economics, management, communication, and sociology. The other area presents a diverse range of fields in the humanities and social sciences that are harder to categorize. The United States (U.S.) and the United Kingdom (U.K.) are prominent, with the U.S. present in all fields and the U.K. in almost all fields. Other countries are more dispersed, with some closer to fields where they have EiCs. Fields like political science, business, and education have EiCs shared across many countries. Fig 2 focuses on the central area of activity, showing connections between countries and fields with five or more EiCs. This includes 23 countries, mostly from Western culture, with exceptions like Japan, China, South Korea, Singapore, and Brazil. The density of connections, especially from the U.S. and the U.K., makes individual connections hard to see.

The average number of EiCs per field is 89.4 (SD = 85.8, range: 5–469). On average, fields are connected to 22% of all possible countries, as shown by the degree (M = 0.223, SD = 0.127, range: 0.042–0.577) in the bipartite network of countries and fields. The closeness centrality indicates that all fields are close to the center of the network and to all countries (M = 0.626, SD = 0.405, range: 0.574–0.753). This indicates that social science disciplines, in general, have geographically diverse connections through their EiCs. This implies that knowledge production and dissemination in these fields is not overly reliant on EiCs from a very limited set of countries. Betweenness centrality (M = 0.014, SD = 0.018, range: < 0.001–0.065) reveals that, on average, 1.4% of the shortest paths go through a given discipline, with the largest discipline (Economics) accounting for 6.5% of all possible shortest paths. This suggests that, in general, social science disciplines do not act as strong gatekeepers in terms of connecting geographically diverse EiCs, while Economics has a more significant role. Fields connected through countries also exhibit a high degree of clustering (M = 0.316, SD = 0.055, range: 0.178–0.410) within their local communities. The average clustering coefficient of 0.316 suggests that, in general, social science disciplines are part of relatively dense tightly-knit communities in terms of the countries where their EiCs are based.

**Fig 3:**
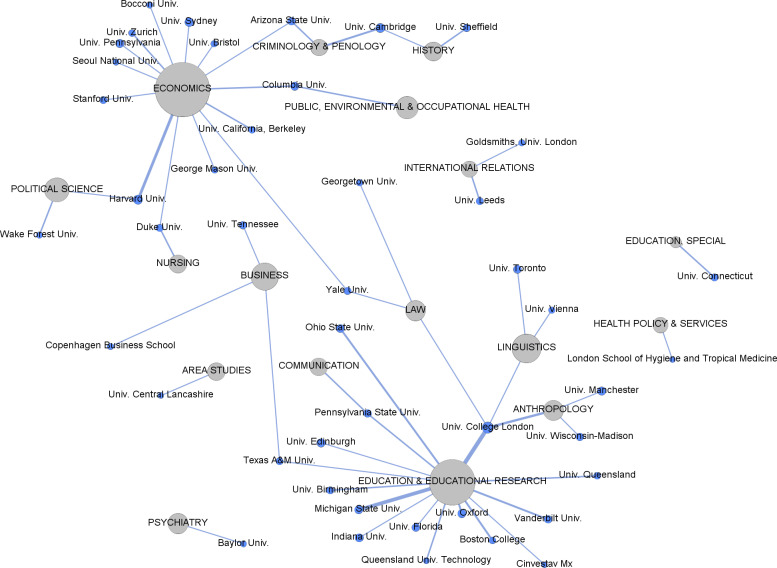
4-slice of the bipartite graph of fields and affiliations, showing only the connections with four or more editors-in-chief. Size of nodes is proportional to the number of editors. Line thickness is proportional to the number of Editors-in-Chief of that field who are affiliated with that specific institution.

Fig 3 presents the slice of the bipartite graph of affiliations and fields. We can see the most influential institutions for the main fields in terms of the number of EiCs that represent them. There is no central institution prevailing across most fields. Influence is evenly distributed among fields with most institutions in the graph only connected to one or two fields. Only the University College London has a strong presence in four fields (law, linguistics, anthropology, and education & educational research). English-speaking institutions from the U.S., the U.K., Australia, and Canada are still prevalent, although other geographies (Italy, South Korea, Switzerland, Denmark, Austria and Mexico) are also represented when we observe the individual fields in the graph. Institutions of these geographies have a significant role in editorial leadership of specific individual fields (economics, business, linguistics, and education & educational research).

Network metrics indicate that the mean degree for fields, as projected through affiliations, is 0.046 (SD = 0.038, range: 0.003–0.201). This suggests that each field establishes approximately 4.6% of the connections with the entire set of affiliations, while the largest fields connect with over 20% of existing affiliations. Closeness centrality is high (M = 0.351) with a low standard deviation (SD = 0.022, range: 0.273–0.402), indicating that most fields are relatively close to each other in terms of shared affiliations. This suggests a certain level of interconnectedness between different fields through the institutions their EiCs are affiliated with. Betweenness centrality (M = 0.031, SD = 0.035, range: < 0.001–0.191) reveals that on average, 3.1% of the shortest paths go through a given discipline, with the largest discipline accounting for 19% of all possible shortest paths through shared affiliations. The high range suggests that several disciplines occupy influential brokerage positions, thereby acting as important intermediaries that connect different parts of the network of fields and affiliations through their EiCs. However, clustering coefficients are low (M = 0.047, SD = 0.015, range: 0.012–0.070), suggesting that disciplines do not establish strong connections with other neighboring disciplines through the affiliation of their EiCs. Fields are not strongly clustered together based on shared affiliations. This suggests that while there are connections between fields through institutions, these connections are not forming tightly knit clusters.

The metrics of top fields of the bipartite graph affiliations-fields are presented in Table 1. The top four fields (Economics, Education and Educational Research, Linguistics, Business) are the same across all metrics. The high degree centrality of these fields suggests they have established connections with a broader range of institutions globally. This can be attributed to several factors including historical global reach for fields like Economics and Business having a long history of international engagement, with established networks in various countries. In addition, the interdisciplinary nature of Education and Educational Research, by definition, intersects with various social sciences, leading to broader institutional representation. Linguistics’ broad applicability research is relevant to diverse disciplines, from language education to computational linguistics, fostering connections with various institutions. Interdisciplinary could also explain that these fields naturally bridge disciplines as showed in their betweenness scores. The high closeness centrality scores of these fields indicate their central position within the broader research network which can be attributed to the influence of their research agendas. Fields like Economics and Business often shape research priorities due to their impact on policy and societal issues. This influence naturally draws collaborations and strengthens their central position in the network. These central fields also tend to attract more funding, prestigious academic positions, and collaborations, further solidifying their influence and closeness to other research areas.

**Table 1:** Top Fields projected through affiliation.

Field	Degree	Field	Closeness	Field	Betweenness
Economics	0.201	Economics	0.402	Economics	0.191
Education & ed. research	0.162	Education & ed. research	0.389	Education & ed. research	0.140
Linguistics	0.110	Linguistics	0.377	Linguistics	0.089
Business	0.108	Business	0.375	Business	0.082
Business, finance	0.088	Business, finance	0.371	Public, env. & occup. health	0.082
Political science	0.086	Political science	0.370	Environmental studies	0.065
Environmental studies	0.084	Environmental studies	0.368	Business, finance	0.065
Public, environ. & occup. health	0.081	Management	0.368	Psychiatry	0.063
Management	0.078	Public, environ. & occup. health	0.368	Management	0.054
Psychology, multidisciplinary	0.076	Psychology, multidisciplinary	0.366	Political science	0.053

Metrics normalized of the bipartite graph fields-affiliations.

Table 2 presents the metrics of top countries for the bipartite graph fields-country. The top nine countries (the U.S., the U.K., Canada, Germany, Australia, China, the Netherlands, Spain, and Italy) underscoring the significant concentration of editorial power within a select group of predominantly English-speaking nations. The high closeness centrality scores of the U.S., U.K., Canada, Germany, and Australia indicate their ability to readily access information and potentially influence knowledge dissemination within the network. This suggests a closely knit community where research trends and collaborations are likely to flow more readily among these nations. The betweenness centrality scores, while still high for these countries, are comparatively lower than their degree and closeness scores. This difference suggests that their influence stems not just from direct connections but also from their role as intermediaries between less connected nations. It is also significant that the U.S. presents a degree of 1 and a closeness of 1, indicating its presence in every single field covered by the JCR. This finding highlights the far-reaching influence of American academics in shaping research agendas across a wide array of disciplines. The role of the U.S. as a central and dominant hub also decreases the distance between countries and fields.

**Table 2:** Top Countries by network metrics.

Country	Degree	Country	Closeness	Country	Betweenness
US	1.000	US	1.000	US	0.103
UK	0.965	UK	0.980	UK	0.093
Canada	0.895	Canada	0.943	Canada	0.078
Germany	0.772	Germany	0.860	Germany	0.051
Australia	0.719	Australia	0.853	Australia	0.048
China	0.684	China	0.824	China	0.038
Netherlands	0.667	Netherlands	0.824	Netherlands	0.037
Spain	0.614	Spain	0.798	Spain	0.029
Italy	0.509	Italy	0.755	Italy	0.021
New Zealand	0.474	France	0.738	Switzerland	0.017

Normalized metrics of the bipartite graph fields-countries.

**Fig 4:**
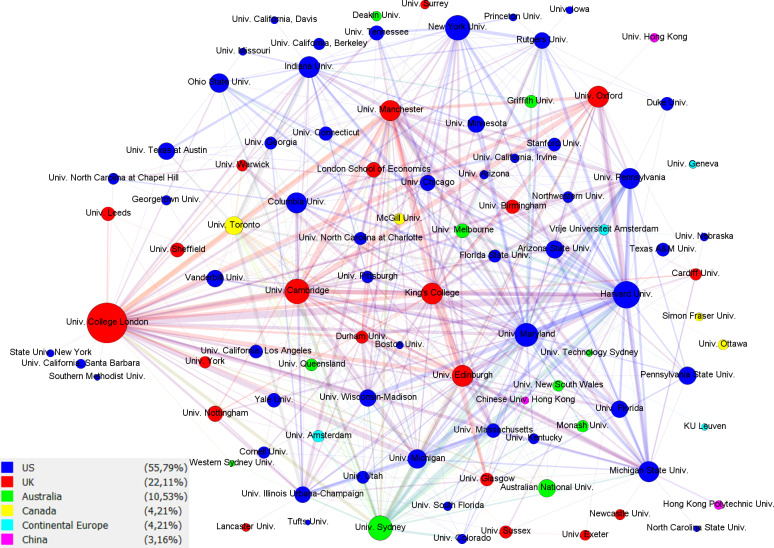
Projection of affiliations by country (Slice). Nodes represent affiliations and are sized proportionally to the number of editors. The thickness of connecting lines represents the number of Editors-in-Chief from those affiliations working in the same fields.

Fig 4 presents a slice of the projection of institutions colored by geography. It represents the central area of the network of EiCs, where connections are determined by shared fields. It highlights the central institutions and how leading editorial responsibilities are distributed globally. We observe that most institutions are from English-speaking countries, although it is difficult to discern central actor or set of actors since many institutions have a significant number of EiCs and create many connections, which also shows that editorial responsibility is distributed among top institutions. Table 3 presents the top affiliations for each network metric. Institutions from English-speaking countries take all positions in the top 10 across all metrics and are predominant in the graph, where only two institutions from continental Europe and one from China are present. It is also significant that although Germany is in the fourth position for all metrics, it has no institutional representation in the figure. This suggests that German influence is distributed among a few smaller-sized institutions in terms of the power they exert through leading editorial board positions. Top institutions in terms of degree are represented by EiCs in many fields pointing to their strength in fostering research across multiple disciplines, attracting scholars working on diverse topics, and contributing to knowledge production in various fields. Top American, British, and Australian institutions are more centrally located within the overall network of social science publishing. This central positioning potentially allows for quicker dissemination of information, greater visibility within the field, and greater potential to influence research trends. Similarly, this group of elite institutions also act as bridges between different clusters of research within the social sciences. They may facilitate the flow of knowledge and ideas between these clusters, fostering interdisciplinary dialogue, and potentially influencing the convergence or divergence of research trajectories. Their high betweenness centrality can also suggest a gatekeeping role influencing the flow of information and resources between different parts of the network. This gatekeeping function might impact which research topics and methodologies gain prominence within those connected clusters.

**Table 3:** Top institutions.

Institution	Degree	Institution	Closeness	Institution	Betweenness
Univ. College London	0.439	Harvard Univ.	0.791	Univ. College London	0.023
Univ. Maryland	0.386	Univ. College London	0.783	Harvard Univ.	0.020
Harvard Univ.	0.386	Univ. Maryland	0.771	New York Univ.	0.019
Univ. Manchester	0.351	Univ. Manchester	0.768	Univ. Maryland	0.017
Univ. Cambridge	0.351	Univ. Sydney	0.756	Univ. Sydney	0.017
Univ. Sydney	0.333	Univ. Cambridge	0.753	Univ. Manchester	0.016
King’s College	0.333	Univ. Michigan	0.745	Univ. Cambridge	0.015
New York Univ.	0.316	Univ. Toronto	0.739	Univ. Michigan	0.014
Univ. Michigan	0.316	Univ. Illinois Urbana-Champ.	0.729	Univ. Illinois Urbana-Champ.	0.013
Univ. Illinois Urbana-Champ.	0.316	Univ. Pennsylvania	0.728	Univ. Edinburgh	0.012

Normalized metrics of the bipartite graph fields-affiliations.

### Gender representation (RQ3)

**Fig 5:**
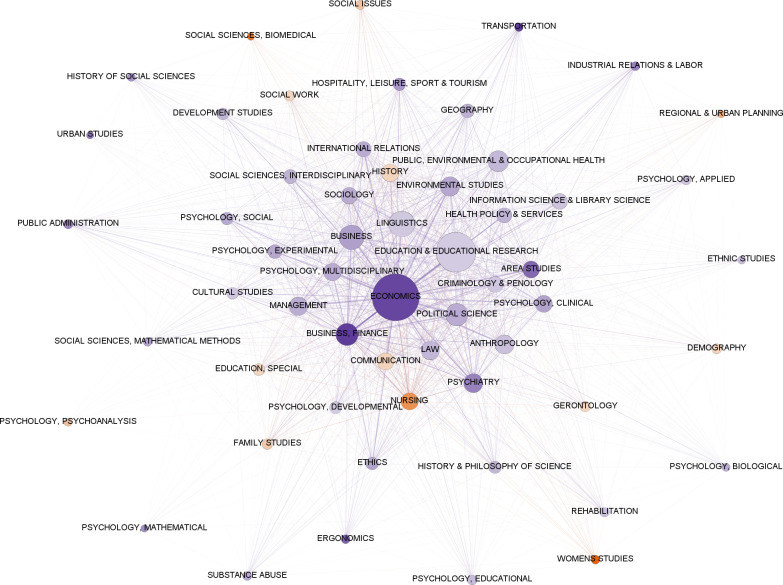
Projection of fields by shared affiliation of Editors-in-Chief. Nodes represent fields and are colored by the predominant gender of their Editors-in-Chief (red - female, blue - male), with darker colors indicating a greater gender imbalance. Node size is proportional to the number of Editors-in-Chief per field. The thickness of a line connecting two fields is proportional to the number of Editors-in-Chief those two fields share from the same institutions.

Fig 5 presents the projection of fields through shared affiliations colored by gender. It shows graphically that male EiCs outnumber female EiCs in most fields. Fields are also highly connected by their shared EiCs, and the density is particularly intense in the center of the figure, where we can also observe that economics, and business and finance present a dark blue color showing that they have a higher proportion of male EiCs. Although the graph shows the gender dynamics and interconnections between different fields of study, we cannot discern any pattern in which fields with a higher proportion of female or male EiCs present more connections to similar fields or to fields showing opposite features. Larger and central fields of the social sciences employ significantly more male than female EiCs. Two-thirds of the fields (38, representing 66.67%) are male-unbalanced, seven are female-unbalanced (12.28%), and twelve (21.05%) are balanced (with a balance ratio of less than 1.2). Female-predominant fields are more related to care and social disciplines, including nursing, social sciences-biomedical, social issues & family studies. Fields showing the highest level of imbalance are business & finance, transportation, and economics with male/female ratios of 4.55, 4.5, and 4.28, respectively, and women’s studies and social sciences-biomedical with female/male ratios of 8 and 4, respectively. We found statistically significant differences for the metrics of affiliations, fields projected by countries, and fields projected by affiliations (Table 4). However, there were no statistically significant differences in countries. Specifically, in affiliations, for degree (U = 189220, p = 0.025) and closeness centrality (U = 178242, p < 0.01), males have significantly higher values compared to females, while for betweenness (U = 179145, p < 0.01) and clustering centrality (U = 186075.5, p = 0.016), females have significantly higher values compared to males. These findings suggest that institutions which employ more male than female editors create significantly more connections to other institutions (degree) and have central positions in the network of affiliations projected by fields (closeness centrality). However, institutions that broadly employ female EiCs have a significantly more relevant role as brokers between affiliations (betweenness) and present a higher level of embeddedness in their local institutional networks (clustering). Regarding fields projected by affiliation, males have significantly higher values of degree centrality compared to females (U = 181, p = 0.046), while for fields projected by country, males also have significantly higher values of closeness centrality compared to females (U = 179, p = 0.016). These findings suggest that fields with more male EiCs create more links to other fields when connections between fields are projected by affiliation, while also occupying central positions in the network when fields are projected by country.

**Table 4:** Gender differences by fields, country, and affiliation for each network.

Metric	Field projected by country	Field projected by affiliation	Country	Affiliation
Degree	U = 185 Z = −1.92	U = 181 Z = −1.99 *	U = 246.50 Z = −1.29	U = 189220 Z = −2.24 *
Closeness Centrality	U = 179 Z = −2.03 *	U = 259 Z = −0.51	U = 247 Z = −1.28	U = 178242 Z = −3.57**
Betweenness Centrality	U = 252 Z = −0.64	U = 259 Z = −0.51	U = 286.50 Z = −0.62	U = 179145 Z = −3.94**
Clustering Coefficient	U = 197 Z = −1.69	U = 284 Z = −0.03	U = 257.50 Z = −1.10	U = 186075.50 Z = −2.42 *

Results of Mann-Whitney U tests with an adjustment for the number of editors as a covariate. * p < 0.05, **p < 0.01.

**Fig 6:**
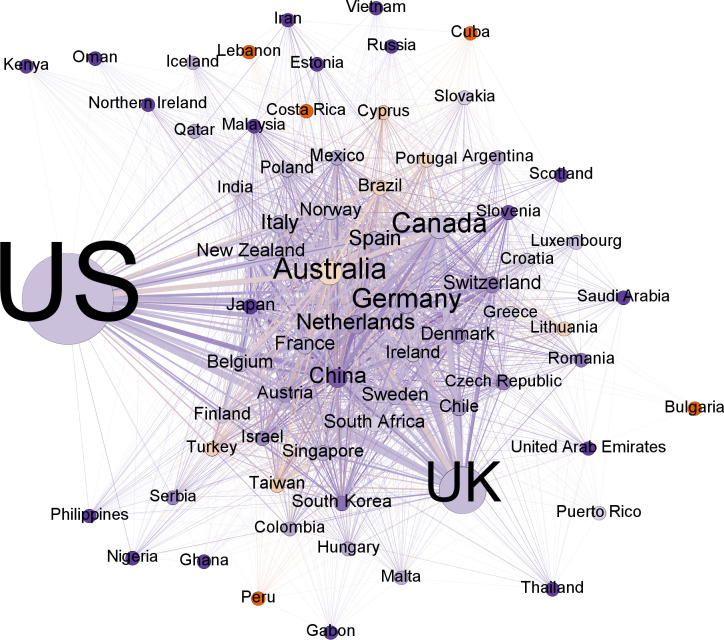
Projection of countries by gender. Nodes represent countries and are sized in proportion to the number of editors. Nodes are colored by the predominant gender of their editors-in-chief (red - female, blue - male), with darker colors indicating a greater gender imbalance. The thickness of connecting lines represents the number of Editors-in-Chief from those countries working in the same fields.

The projection of countries colored by gender (Fig 6) provides an overview of gender representation in different regions showing graphically that most countries have more male than female EiCs. We can observe the two poles of geographic influence, the U.S. and the U.K., that we previously observed. In terms of gender representation, we cannot find any pattern of connections or aggrupation between fields with higher proportions of female or male EiCs. The average male/female ratio for all countries is 1.67, which is close to the ratio of the complete dataset (1.61). Thirty-six countries (76.60%) are male-unbalanced, four are female-unbalanced (8.51%), and seven (14.89%) are balanced (with a balance ratio of less than 1.2). The countries representing the highest level of imbalance in their EiCs are Japan, with a ratio male/female of 5.5, and China with a ratio male/female of 4.14. All top countries, except Australia, present substantially more male than female EiCs (Table 5). Australia presents a ratio very close to one. Further, by examining all countries with 20 or more records in the dataset, we do not find any in which the number of women outweighs men by more than 20%. There are no statistical differences, though, for any of the network metrics between male- and female-unbalanced countries. This is probably because of the sample presents a low number of countries in which women outweigh men.

**Table 5:** Gender balance of top countries.

Country	#editors	Male/female ratio
US	1951	1,47
UK	830	1,63
Australia	271	−1,02
Canada	230	1,52
Germany	216	2,07
China	120	4,14
Netherlands	120	2,64
Spain	100	1,86
Italy	92	1,76
France	68	1,83
Switzerland	56	3,31
Sweden	53	1,89

Countries with more than 50 editors are included.

**Fig 7:**
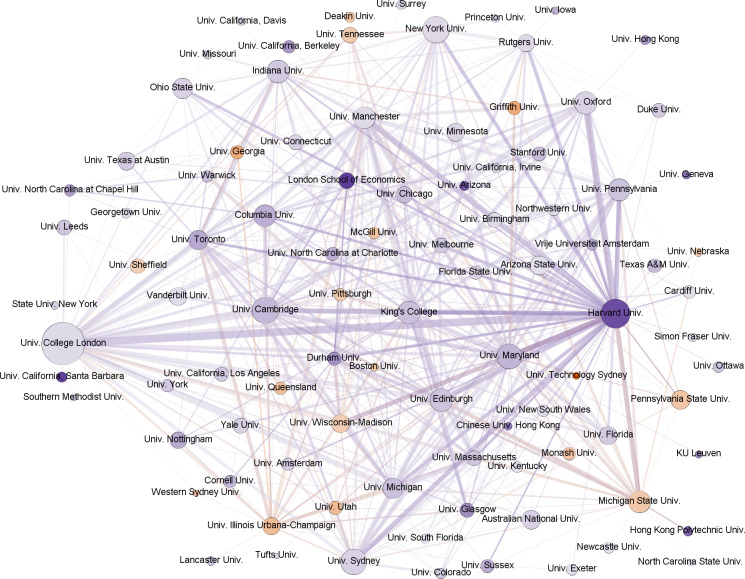
Projection of affiliations by gender (Slice). Nodes represent affiliations and are sized proportionally to the number of editors. Nodes are colored by the predominant gender of their editors-in-chief (red - female, blue - male), with darker colors indicating a greater gender imbalance. The thickness of connecting lines represents the number of editors-in-chief from those affiliations working in the same fields.

Fig 7 presents the projection of the slice of institutions colored by gender. Again, we find more institutions with male EiCs than with female EiCs, and we cannot see any pattern of connection or aggrupation between institutions with higher proportions of female or male EiCs. We can see graphically in the slice represented in the graph that Harvard University and the London School of Economics show the highest level of imbalance among the top institutions, while in proportion, Australia presents more cases of universities with a higher proportion of female EiCs. Sixty institutions (63.16%) present a substantial male imbalance, 15 institutions (15.79%) present substantially more female EiCs, while 20 institutions (21.05%) present a balance ratio of less than 1.2. The institutions presenting a higher level of imbalance at the core are the London School of Economics, the University of California-Santa Barbara, and Harvard University, with male/female ratios of 5.67, 5.5, and 5.17, respectively. Several institutions also present substantially more female EiCs. At the top, we find the University of Technology Sydney, Griffith University, and the University of Georgia with female ratios of 5.67, 2.4, and 2.4. The average balance ratio for the complete set of institutions (N = 1,485) is 1.61, which is larger than the average of 1.29 found in the slice representing the core (N = 95). This suggests that the balance gets worse in the periphery for institutions with fewer overall EiCs and influence.

## Discussion

As mentioned earlier, existing literature highlights the critical role of editorial boards in shaping and disseminating scientific knowledge. It also examines the dynamics of representation among editorial board members, focusing on aspects such as gender, geographical diversity, and institutional affiliations within specific disciplines or across multiple fields. Since past studies are mainly restricted to observing the representation composition of journal editors based on single or interlocking fields, our study expands the empirical research on this subject by offering a robust investigation into editorial leadership in social sciences journals in terms of gender, fields, geographical, and institutional networked connections, which will be discussed in the sections below.

In line with our main findings and conclusions from previous studies on the composition of journal authorship and EiCs, we found clear evidence for the concentration of editorial authority in two English-speaking countries: the U.S. and the U.K. Therefore, our aim is to contribute to scholarly discussions on diversity, equity, and inclusion (DEI) within academia by revealing the existing dominance of male EiCs from U.S. and U.K institutions and in social sciences journals, and the need to create an even balance in editorial leadership, keeping current levels of scientific excellent constant. As outlined throughout the study, we do not advocate for increasing diversity at the expense of scientific excellence. Instead, we emphasize the importance of remaining open to understanding epistemic traditions that may offer valuable perspectives. From an economic standpoint, it stands to reason that countries investing the most in science tend to dominate the most prestigious journals. However, it is equally true that the marketplace of ideas should remain accessible to all, provided that the published work adheres to certain standards of research excellence.

### Connections between fields and representation of editors-in chief across institutions

Our analyses reveal the network structure of EiCs across different institutions and disciplines by affiliations. Specifically, we can see differences in the predominant institutions for each discipline, which include the lack of diversity in the gender of the EiCs and geographical dominance of U.S. and U.K institutions. At a descriptive level (Table 1), we found that the notable fields by affiliation were Economics, Education & Educational Research, Linguistics, and Business across all metrics, particularly for their degree and betweenness centrality. This implies that these fields have EiCs from a wider variety of institutions compared to other fields (degree), while they are also more likely to act as intermediaries between institutions with less direct connections (betweenness). This could indicate they have a greater potential to influence the flow of knowledge and resources within the broader network of social sciences. Findings then imply that these fields hold a more influential position within the network of social sciences publishing. This raises important questions about potential bias due to concentrated influence, possible advantages in resource allocation and barriers for other fields. Therefore, it can be assumed that, to a very large extent, there is a concentration of editorial leadership and authorship in social sciences journals among the referenced disciplines in the dominant English-speaking institutions. As such, this may privilege the knowledge repository of the affiliated disciplines, regardless of their scientific merits, and creates a highly skewed global scholarship where less dominant scientific discourses are unable to compete [38].

Another important finding is the similarities of each dominant field to a certain group of countries’ top institutions, mainly emphasizing epistemic, critical-cultural, and topical scientific issues in Euro-American hegemony [39] with the U.S. and U.K. institutions, showing also prominent results across all network metrics (Table 3). Institutions like University College London, Harvard University, and University of Maryland consistently rank high in degree centrality. This suggests these institutions have a broader reach within the network, collaborating with a more diverse range of institutions through their EiCs in many fields. This wider network of connections could enhance their influence and impact within the field. Similarly, the high closeness centrality scores indicate that these institutions are positioned closer to the center of the social sciences publishing network. They have shorter paths to other institutions, which might give them an advantage in terms of accessing information, disseminating their research, and shaping academic discourse. The prominent betweenness centrality scores also suggest that they occupy influential brokerage roles within the network. Their position allows them to connect different parts of the social sciences publishing landscape and potentially influence the dissemination of research and ideas across various subfields. This indication has a strong likelihood of reinforcing “the prestige bias” in favor of affluent Western institutions and might serve as a prerequisite for the selection of editorial boards, which would then impact the norms and values of respective journals.

In a similar vein, the results depicted in Fig 3, which represents the slice of the bipartite graph of fields and affiliations, affirm an even distribution at the core, with no single institution or set of institutions exerting central dominance across fields; nonetheless, there is a substantial presence of U.S. and U.K. institutions in top individual fields like Economics, Political Science, Education, Linguistics, and Business, and their interconnectedness to other disciplines. For example, University College London has a strong presence in four disciplines. Likewise, Fig 4 deconstructs the predictions of institutional affiliation by country, and it discloses notable affiliations of EiCs in the U.S. and the U.K., which is consistent with our earlier results.

Some studies have examined the overrepresentation of U.S. and U.K. institutions among EiCs across many fields, portraying the dominance of English-speaking countries in the academic setting. [32], who investigated journals listed in the Web of Science’s databases to provide a comprehensive picture of the geography of EiCs, found that most EiCs are located in countries of the Anglosphere, predominantly the U.S. and the U.K. The author notes that a significant number of academic publishers and professional organizations that publish scholarly journals are based in both countries where most EiCs are also based. In a study examining the role of editorial board members as gatekeepers in science, Baccini and Re [34] discovered that editorial boards of economics journals, and consequently the field of economics, are dominated by U.S.-based scholars and they are mostly affiliated to elite universities. Therefore, the authors suggest that it is essential to implement measures aimed at reducing this concentration to promote pluralism and diverse perspectives within the discipline.

In relation to our study, our findings raise concerns about how Western subordination might shape research agendas and the inclusivity of different viewpoints, particularly those from institutions in non-English-speaking countries, which are notably underrepresented in scholarly publications [40] and, as we establish in this study, in leadership roles too. In general, the implication of these results calls for “decentralization, differentiation, and pluralist thinking” [23: 2] in the academic community and a need to broaden the intellectual and geographical scope of editorial decision-making across disciplines and institutions so that regions other than the Western world (or the Anglosphere) are effectively represented.

In addition, since this study investigates the network distribution of EiCs and the EiC is distinct from other editorial board positions, the EiC position must be filled by a renowned researcher in each field who is arguably affiliated with a high-ranking university to potentially help increase the journal’s prestige and protect the quality of published works, while still encouraging diverse analytical interrogation, robust validity of claims in each field, and global hegemony [41,42]. Hanitzsch [38] expressly states that academic fields with more institutional, cultural, and geopolitical diversification in the editorial boards will encourage greater resistance to global crises and augment universal and evolutionary scientific debates.

### Geographical composition of journal editors-in-chief

As observed in previous studies [6,10], editorial boards for social sciences journals are not only prevalent in elite Western institutions but mostly represent geographies like the U.S. and the U.K. in selected network metrics (degree, closeness, and betweenness). Our findings support these conclusions by unraveling two main poles represented by two countries—the U.S. and the U.K. The links of these geographies (Figs 1 and 2) cast a long shadow across all or nearly all fields, suggesting a concentration of editorial authority in these countries, which may eventually influence the decision-making process of scientific publishing in almost all fields.

While there are other countries with less visibility in each field of study, such as Canada, Germany, Australia, China, the Netherlands, France, and Spain, the prominence of institutions from non-English-speaking or minority countries is notably insufficient. This is particularly true for top positions and central roles within the network (Table 2). This lack of visibility likely leads to a significant shortfall in the recognition of empirical knowledge and editorial leadership from these nations, which plays a crucial role in shaping the future of sciences. This insinuates that efforts to foster diversity in academia and editorial boards may be hindered by the geographical dominance of a few affluent nations at the helm of academic decision-making.

Additionally, Fig 2 illustrates the network density created by the two main actors—the U.S. and the U.K.—along with the sizable connections of less prominent countries like Australia, South Africa, and Germany. These countries, together with several others, share five or more EiCs across JCR fields, predominantly representing Euro-American cultures with a slight Asian influence. Thus, the West remains in control of the structure of knowledge propagation and leadership in scientific journals [43]. As a matter of fact, this may exacerbate the disparities that Waisbord [41] and Demeter [44] describe as global academic knowledge production inequalities, specifically in communication studies, where the US and a few other European countries form the powerful center, while the rest of the world accounts for between 1% and 5% of knowledge in the field.

### Gender composition of journal editors-in-chief

We found clear evidence for the uneven distribution of EiCs based on gender differences, such that the typical journal mainly comprises male scholars or EiCs from elite Western universities, and this supports previous findings of the lack of female presence as editorial board members, despite the recent improvement in the presence of female authorship in specific fields and journal networks [13]. To explore gender diversity among EiC, we compare female presence across affiliations, countries, and disciplines. Our statistical analyses in Table 4 prove that gender imbalance is significant across all metrics in the network of affiliations, and for closeness centrality in fields projected by affiliation and degree for fields projected by country. We did not find any difference for the interconnected network of countries. The significant gender differences in degree and closeness centrality highlight the over-representation of male scholars in fields and institutions with more connections, playing a central role in their network. This over-representation can potentially impact research agendas, publication biases, and the dissemination of knowledge. The dominance of male EiCs in central network positions might affect the diversity of research perspectives and the overall direction of scholarship, potentially leading to a narrower scope of published research. The higher degree and closeness centrality values for male EiCs in affiliations and fields suggest that these are more likely to be in positions that facilitate extensive networking and influence within academic publishing. However, the higher betweenness centrality and clustering values for institutions with a majority of female EiCs indicate that they may play a unique role in fostering interdisciplinary collaborations and bridging different research communities. Female EiC institutions appear to maintain close ties with their neighboring institutions in the network, which could facilitate more cohesive and collaborative research environments. This unique positioning might enable female EiCs to act as crucial connectors within the academic network, promoting a more integrated and inclusive research landscape. These findings underscore the importance of considering gender dynamics in academic leadership roles. The differential centrality measures between male and female EiCs suggest that gender diversity in editorial positions can influence the structure and dynamics of academic networks. Promoting gender diversity in these roles could enhance the inclusivity and breadth of academic research, fostering a more balanced and representative scholarly community.

Similarly, the results demonstrated in Fig 5 show graphically that all research fields are dominated by male EiCs, except for nursing and women’s studies, and with a lower ratio but higher prevalence of women compared to men in areas like family studies, psychology-psychoanalysis, communication, special education, history, biomedical social sciences, regional and urban planning. This suggests gender role stereotyping in academic journals, as females are more noticeable in social care, health, communication, sociology, and psychology, typically tagged as feminine research fields and aligning with societal gender norms [10,45,46]. Indeed, the overall projection of males outnumbering female EiCs seem to perhaps reinforce the lack of female presence, thereby confirming the observation from past studies [47,48]. Further, this phenomenon illustrates a systematic bias that could influence publication trends, editorial choices, and academic discourse in general. Gender disparity may also shape research agendas, potentially perpetuating a skewed perspective in various disciplines [42].

While our analyses did not delve into the mechanisms involved in the inequitable differences we found in the gender composition of EiCs, a couple of recommendations could address this issue. As mentioned in Mauleon et al [49], a possible approach is an increase in the proportion of women scientists as first authors or co-authors in top papers, especially in fields where males predominate. This could serve as a useful measure to foster academic recognition for women and promote gender diversity on editorial boards. Another potential intervention is a greater propensity of academic journals to involve more expert female scholars in editorial decision-making, which could contribute to the diversification of gendered perspectives in social sciences and lead to the long-term advancement of female EiCs in academic rank.

Concerning institutional differences, it should be noted that leading or Ivy League institutions in the U.S. and the U.K. represent a higher number of editorial boards with predominantly male scholars at varying densities. In parallel, our interpretation of the graph presented in Fig 7 indicates that a small number of U.S. institutions and very few European institutions have more female representation. While our findings show the prevalence of women EiCs in certain institutions, the numbers are still evidently lower compared to their male counterparts across other affiliations.

Descriptive analyses in Table 5 reveal that male EiCs are also dominant in top countries, with higher ratios of males in all except Australia. Additionally, this pattern is reflected in almost all nations in our findings (Fig 6), except smaller and non-central ones engaged in scholarly production, such as Cuba, Lebanon, Bulgaria, Costa Rica, and Peru, which present clear female representations. In comparison to the main pattern, which constitutes more male editorship and in significantly larger numbers, a few other nations, mainly represented by Australia and some European geographies, have a somewhat balanced gender representation among EiCs. Thus, one of the recommendations arising from our findings as a strategy to address male predominance in the global academic environment is the need to dissipate the monopoly of male EiCs in top Western countries, facilitate further decentralization of editorial authority across less dominant nations, and support a significant increase in the inclusion of female researchers/scientists in journal editorial boards as well as in the byline of publications.

### Limitations and Conclusion

Although this study successfully examined the web of institutional, geographical, and gender connections within the editorial leadership in social scientific journals, it has some limitations. First, the focus on JCR-ranked journals as a standardized measure may introduce a bias toward other established and well-known academic journals. The risk of prejudice could impact the generalizability of the findings, particularly considering the number of social sciences journals that may not be included in the JCR rankings.

Second, the study focuses on the JCR ranking system, which heavily favors indexing English-language journals and overlooks publications in other languages. This likely contributes to the observed dominance of Anglo-American scholars and institutions, as many journals in non-English languages are left unnoticed. While JCR ranking remains a crucial tool for assessing journal significance and evaluating scholars and institutions in academic settings, future research should consider utilizing alternative ranking systems like Scopus, which offers greater diversity by including journals published in national languages. This would provide a more comprehensive analysis of the editorial leadership landscape across different linguistic and cultural contexts in the field of social sciences. Additionally, future research could complement the data with other methods, such as interviews or surveys with editors and publishers, to gain deeper insights into the factors influencing editorial governance within diverse linguistic and cultural settings.

Third, while the study identifies gender imbalances, it may benefit from a deeper qualitative analysis to understand the underlying factors contributing to these imbalances. A deeper exploration of the experiences and challenges faced by EiCs, particularly female EiCs and non-EiCs, could provide insights into the mechanisms perpetuating gender disparities in editorial authority.
